# Evaluation of Bony Femoral Morphological Parameters in Anterior Cruciate Ligament Injury Using Magnetic Resonance Imaging: A Retrospective Unmatched Case-Control Study

**DOI:** 10.7759/cureus.55463

**Published:** 2024-03-03

**Authors:** Sagar Maheshwari, Joel Thomas, Rajesh Kuber, Rahul Arkar, Purnachandra Lamghare, Madhuree Avhad, Thulasi Tharmalingam, Karen Abraham, Amit Kharat, Dhammapal Bhamare, Julie Thomas

**Affiliations:** 1 Radiology, Barking, Havering and Redbridge University Hospitals NHS Trust, London, GBR; 2 Radiology, Dr. D. Y. (Dnyandeo Yashwantrao) Patil Medical College, Hospital & Research Centre, Pune, IND; 3 Musculoskeletal Radiology, Dr. D. Y. (Dnyandeo Yashwantrao) Patil Medical College, Hospital & Research Centre, Pune, IND; 4 Radiology, Datta Meghe Institute of Higher Education & Research (Deemed to be University), Wardha, IND

**Keywords:** anterior cruciate ligament (acl), knee ligaments, anatomical factors, notch width index, knee mri, acl tear

## Abstract

Background

Over time, there has been a noticeable increase in anterior cruciate ligament (ACL) injuries. The current imperative is to anticipate predisposing factors and proactively prevent ACL injuries. The occurrence of ACL injuries has been linked to diverse factors associated with the morphology of the distal femur.

Objectives

Through this study, we aim to compare the anatomic variables of distal femur morphology such as notch width (NW), bicondylar width (BW), notch entrance width (NEW), and notch width index (NWI) between patients with ACL injuries and non-injured patients using MRI. We also aim to make a comparison of these factors between male and female genders to assess the gender variability.

Material and methods

A retrospective case-control study was conducted amongst patients who underwent MRI Knee scan for clinical suspicion of internal derangement during the study period. We selected the first 125 individuals who were found to have ACL injury in the MRI scans and selected another 125 individuals who had an intact ACL in the scans, to serve as controls in the study. Demographic information was retrieved from the hospital's electronic records, and the assessment of NW, NWI, BW, and NEW was conducted through a review of MRI sequences. They were then compared between the cases and control groups, as well as between male and female genders.

Results

The ACL-injured group exhibited statistically significant reductions in NW and NWI. While 17.39 mm was the mean NW among cases, 17.86 was the mean value among controls. Similarly, the mean NWI was 0.25 among patients with ACL injuries and 0.27 among controls. Gender-based comparisons also revealed statistically significant differences in NW and NWI measurements, where females were reported to have comparatively lower measurements. The mean NW for males and females in the injured group were 18.26 mm and 15.40 mm, respectively, while it was 18.71 mm and 16.90 mm, respectively, in the control group. In the case of NEW, males in the injured group had a slightly higher value (21.33 mm) than the controls (20.65). Females on the other hand exhibited a lower mean value of NEW in ACL-injured group (18.51 mm) in comparison to the non-injured (18.79 mm). BW did not seem to show a significant difference between the two groups.

Conclusions

In the studied population, ACL injuries demonstrated a higher occurrence in individuals with a narrow femoral intercondylar NWI. If any of these characteristics are identified in an MRI, it may be helpful to identify individuals who are at a higher risk of developing ACL injuries and may thereby help in planning preventative strategies.

## Introduction

The anterior cruciate ligament (ACL) is the most commonly injured knee ligament, running from the medial aspect of the lateral femoral condyle to the medial tibial eminence and traversing the intercondylar fossa. The primary role of the ACL is to restrict anterior translation. Additionally, it plays a preventive role in restraining lateral rotation, varus and valgus stresses, extension, and hypertension [1]. Magnetic resonance imaging (MRI) is the preferred imaging method for assessing knee and ACL injuries due to its proven high sensitivity and specificity [2].

With the recent innovations in surgical instruments and advanced surgical techniques, ACL reconstruction has been widely practiced worldwide. [3-5]. Nevertheless, the escalating expenses associated with reconstructive surgeries and the often persistent instability following the procedure emphasize the importance of implementing preventive measures against ACL injuries. Several factors have been suggested to play a role in the onset of ACL injuries, categorizable into two main types: intrinsic and extrinsic factors. Intrinsic factors encompass anatomical elements (e.g., narrow femoral intercondylar notch width (NW)), gender, previous injuries, and more. On the other hand, extrinsic factors involve environmental influences like the friction between players' shoes and the ground, BMI, and similar considerations [6]. Certain individuals face a higher likelihood of experiencing an ACL tear than others. Overall, women tend to face a greater risk of injury compared to men, and statistical analyses reveal that the volume of the femoral notch is smaller in women than in men [7-10].

Palmer et al. in 1936 were the first to suggest that narrowing of the intercondylar notch was a risk factor for ACL injury [11]. Following Palmer's research, numerous studies have been undertaken to explore the influence of the intercondylar notch and other bone morphological parameters on ACL injuries. While several studies have underscored the significance of a narrow NW in relation to ACL injuries, there is still no unanimous consensus on this theory [12-16].

The objective of this study was to compare the femoral bony morphology of ACL-injured and non-injured individuals using MRI scans. We measured anatomical variables of the distal femur and compared these parameters between the two groups to ascertain whether they serve as anatomical risk factors for ACL injuries. We also aimed to assess if the gender predisposition was valid amongst the Indian population as well.

## Materials and methods

Study overview

We performed a retrospective unmatched case-control single-centre study on 250 patients who had MRI Knee done in Dr. D. Y. Patil Medical College Hospital and Research Centre, Pune, India, between January 2018 and August 2019. This method of study was chosen as it was found to the best in investigating an association between specific risk factors and a particular outcome.

Ethical approval

This study was approved by the Scientific and Ethical Committee of Dr. D. Y. Patil Vidyapeeth, Pune, India (approval number: DYPV/EC/290/2019). Since this was a retrospective study with anonymous data analysis, it was deemed exempt from requiring informed consent from the patients.

Study criteria

This study involved subjects who underwent MRI Knee on suspicion of any internal derangement during the study period. We looked at the MRI scans of 250 patients who were divided into two groups. One group consisted of 125 patients with ACL injury diagnosed on the MRI scan while the other group consisted of an equal number of patients whose ACL remained intact on the scan and hence served as the control group. Non-injured patients were identified as those with intact anteromedial and posterolateral bundles exhibiting normal signal. Patients with ACL injuries were characterized by abnormal signal and fibre discontinuity, leading to partial or complete tears. All types of ACL injuries such as sprain, partial/complete tear, including avulsion injuries, were considered. Exclusion criteria included individuals with any knee pathology apart from ACL injuries such as known knee deformities, prior knee surgery, and inflammatory, degenerative, and septic arthritis. The purpose of the exclusions was to ensure that the study subjects were free from conditions that already compromised the integrity of the ACL and the anatomy of the joint. The study included patients aged between 18 and 50 years, taking into account skeletal maturation and aiming to avoid degenerative changes that might be common in the age group not covered by the study.

Study procedure, MRI technique, and measurement

**Figure 1:**
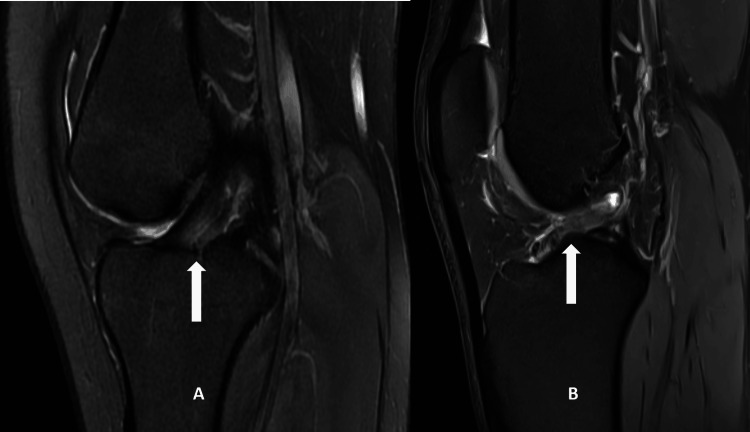
Comparison of a normal ACL (A) and an injured ACL (B) ACL: anterior cruciate ligament

**Figure 2:**
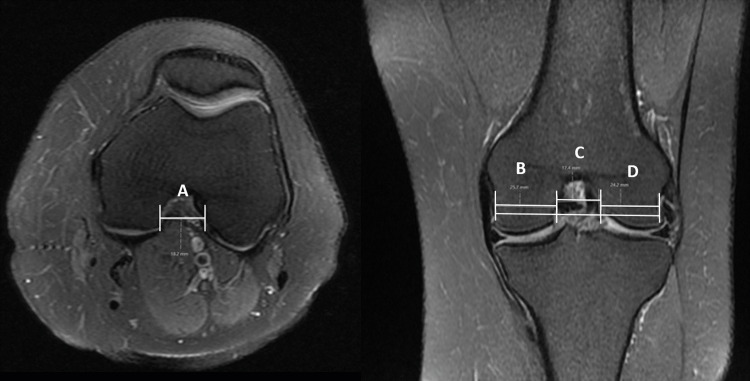
Femoral measurements: Notch entrance width (A) measured on axial image; Notch width (C) and bicondylar width (B+C+D) at the level of popliteal groove measured on coronal images.

Patient demographics and available clinical history were gathered from the electronic hospital patient information databases. All the study subjects underwent a standard knee MRI on a 1.5 T MAGNETOM® Avanto (Siemens Healthineers, Erlangen, Germany) and 3T MAGNETOM Vida (Siemens Healthineers). Before beginning the trial, the MRI protocol's suitability for diagnosing ACL damage was reviewed, modified, and brought up to standard. All images were taken with the patient supine and their knee flexed at a 10-degree angle. MedPac Systems picture archiving and communication system (PACS) imaging software (Medpac System Solutions India LLP, Bengaluru, Karnataka, India) was used for assessing the various morphometric parameters. Multiplanar T1 and fluid-sensitive sequences were used to diagnose ACL injury (Figure 1) and assess various measurements of femoral NW, bicondylar width (BW), and notch entrance width (NEW). Notch width index (NWI) was calculated by dividing NW by BW, where both were measured at the level of the popliteal groove (Figure 2).

For the measurements, initially, a reference line was marked from one condylar end to the other, spanning across the most distal aspect of the femur. The measurement of femoral BW was taken along a line parallel to the reference line, passing through the popliteal groove. NW was determined by measuring the distance from the medial articular cartilage margin of the lateral femoral condyle to the lateral articular margin of the medial femoral condyle while NEW was measured in the axial plane at the level of appearance of posterior condylar articular cartilage. We compared the parameters between the cases and controls and then also performed subgroup analysis based on gender.

Statistical analysis

Epi Info™ statistical software (Centers for Disease Control and Prevention, Atlanta, Georgia, United States) and MS Office 2016 (Microsoft Corporation, Redmond, Washington, United States) were used for data analysis. The measurement data were expressed as the mean ± standard deviation (x̄ ± SD), while numbers or percentages were employed for the representation of categorical data. Summaries and tabular representations were utilized as needed. The association between two categorical variables was examined using the Chi-square (χ2) test. To assess differences in the means of analytical variables between independent groups, the unpaired t-test was employed. Statistical significance was attributed to results if the calculated p-values were below 0.05.

## Results

**Figure 3:**
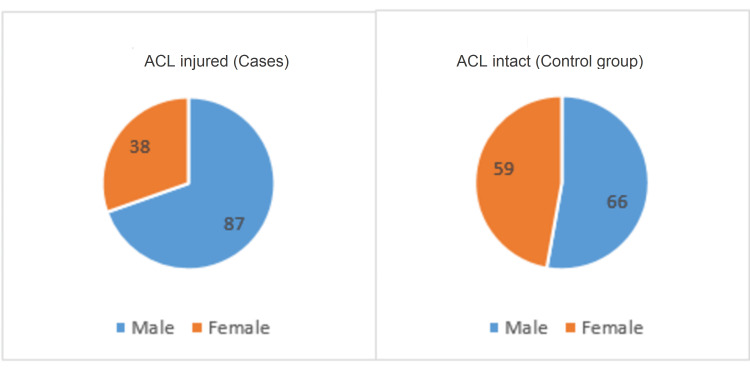
Graphical representation of distribution of patients based on gender

The study was conducted among 250 patients of which 125 showed ACL injuries on MRI and were considered the "cases" group and the remaining 125 patients were the "control" group as they were noted to have intact ACL on the MRI. The cases group (n = 125) comprised 87 males and 38 females, while the control group (n=125) included 66 males and 59 females (Figure 3). The mean age of men in the ACL-injured group was 30.7 years while 37.5 years was the mean age in females. In the control group, 35.8 and 33.8 years were the corresponding mean ages of males and females, respectively.

**Figure 4:**
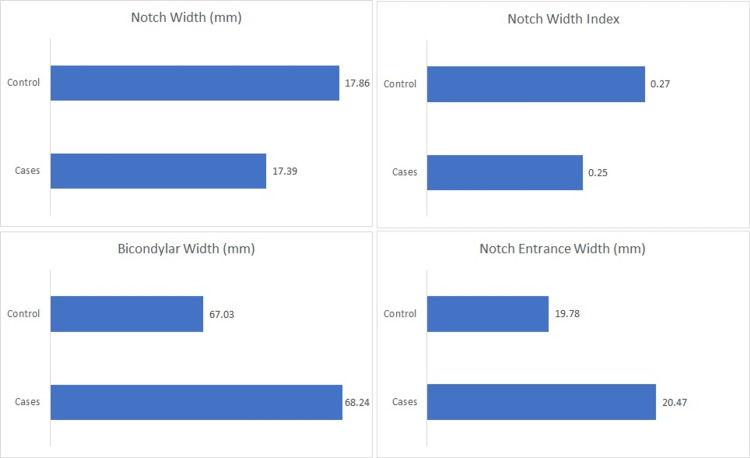
Graphical representation depicting variation of the various parameters between cases and controls

Table 1 shows the mean values of the various bony morphological features of the knee being compared between the two groups. The ACL-injured group had a smaller NW of 17.39 mm in comparison to the control group which had a mean NW of 17.86. Similarly, the mean NEW as well was lower amongst the ACL-injured group (0.25 vs 0.27). In contrast, the BW was slightly higher amongst the cases with a mean value of 68.24 mm in comparison to the controls with a mean value of 67.03. NEW similarly remained greater among the ACL-injured group than the controls (20.47 mm vs 19.78 mm) (Figure 4).

**Table 1:** Comparison of bony morphological parameters of the knee between ACL injured (cases) and ACL non-injured (controls) ACL: anterior cruciate ligament

Mean Values	Cases	Controls
Notch width (mm)	17.39	17.86
Bicondylar width (mm)	68.24	67.03
Notch width index	0.25	0.27
Notch entrance width (mm)	20.47	19.78

Statistical analysis was performed based on the gender of the subjects as well (Table 2). Generally, as expected, all four anatomical variables were slightly lower among the females in comparison to men. The mean NW was found to be comparatively lower in the ACL-injured group among both males and females. The mean NW of men in the cases group was 18.26 mm while in the controls, it was 18.71 mm. Similarly, females had a slightly lower value (15.40 mm) among the cases in comparison to the controls (16.90 mm). Both genders generally showed lower values of NWI in the group with ACL injuries, with females having a lower value (0.24) in comparison to males (0.25). BW was found to be similar in both the injured and non-injured groups (70.95 mm and 70.93 mm, respectively) in males and (62.04 mm and 62.62 mm, respectively) in females. NEW showcased a slightly different picture. Males with ACL injury were found to have a greater value of NEW (21.33 mm) as compared to the controls (20.65 mm). However, females had a slightly lower value amongst the cases (18.51 mm) in comparison to non-injured (18.79 mm). 

**Table 2:** Gender-based bony morphologic differences between ACL injured (cases) and ACL intact (controls) patients ACL: anterior cruciate ligament

		Cases				Controls		
	Gender	Mean	SD	p-value		Mean	SD	p-value
Notch width	Male	18.262	2.074	< 0.001		18.716	1.997	< 0.001
	Female	15.407	1.678			16.909	1.790	
Bicondylar width	Male	70.950	3.556	< 0.001		70.936	4.651	< 0.001
	Female	62.045	3.562			62.650	4.098	
Notch width Index	Male	0.257	0.025	0.071		0.264	0.023	0.146
	Female	0.248	0.025			0.270	0.023	
Notch entrance width	Male	21.330	2.908	< 0.001		20.656	2.743	< 0.001
	Female	18.516	2.472			18.794	2.531	

The odds ratio of NW was 1.43 (95% CI, 1.202-1.701), which implies that there is a 1.43-fold increase in the odds of getting an ACL injury with a reduced NW. On the other hand, BW and NEW showed an odds ratio of 0.907 (95%CI, 0.849-0.969) and 0.883 (95% CI, 0.789- 0.987), respectively (Table 3).

**Table 3:** Logistic regression association between bony femoral morphological characteristics on MRI knee with ACL injury ACL: anterior cruciate ligament

	B	Standard Error	P-value	Odds Ratio	95% CI	
					Lower	Upper
Notch width	0.357	0.089	<0.001	1.43	1.202	1.701
Bicondylar width	-0.097	0.034	0.004	0.907	0.849	0.969
Notch entrance width	-0.125	0.057	0.029	0.883	0.789	0.987

**Figure 5:**
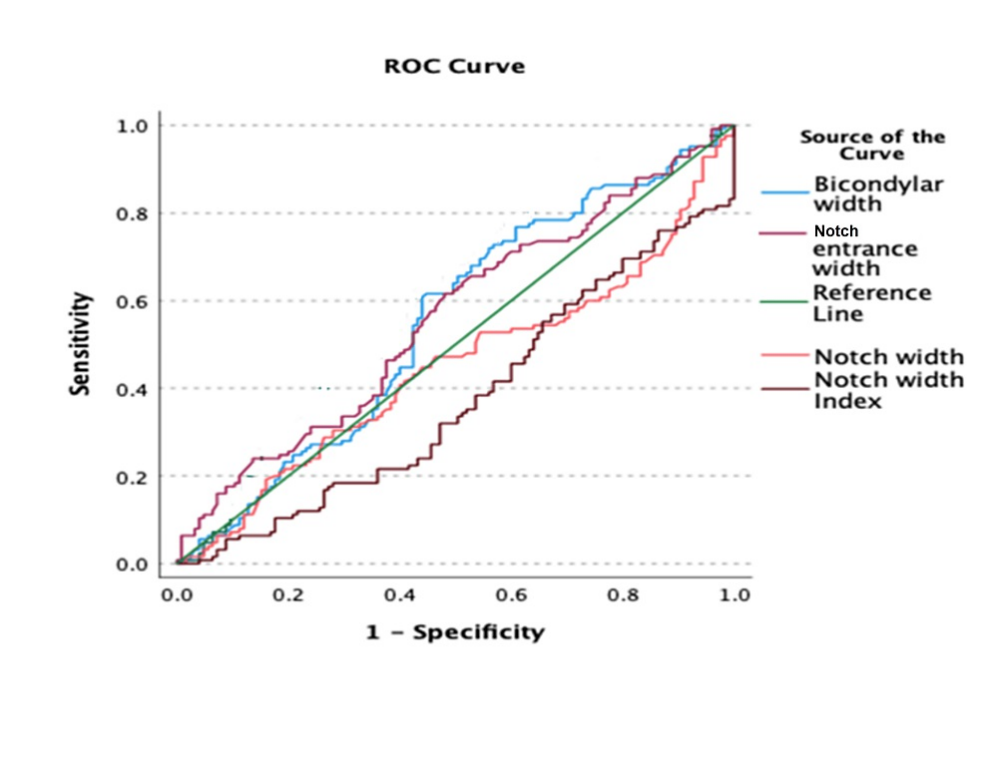
ROC curve of various parameters in predicting ACL tear ROC: receiver operating characteristic; ACL: anterior cruciate ligament

Receiver operating characteristic (ROC) curves were employed to assess the parameters associated with predicting ACL tear injuries (Figure 5). The analysis revealed a positive correlation between BW and NEW, while NW and NWI exhibited a negative correlation. The area under the curve (AUC) for BW, NEW, NW, and NWI were 0.553, 0.561, 0.447, and 0.374, respectively.

## Discussion

The ACL plays a vital role in stabilizing the knee joint and contributes significantly to other key functions within the knee. A very common mechanism of injury is an exertion of varus and internal rotation force on the tibia that occurs on hyperextension of the knee [17]. Our study aimed to evaluate and establish the correlation between the likelihood of ACL injuries and specific parameters of the distal femur observed on MRI, including NW, NWI, BW, and NEW. Additionally, we examined gender variations in this context.

Various studies have been conducted across India to assess the causative anatomical factors, similar to our study, by utilizing MRI scans and radiography [17-20]. There exists a notable lack of consistency in findings on the true predictive value of the NWI for ACL injury. While some studies suggest a link between a narrower NWI and a higher likelihood of ACL injuries [21,22], studies by Teitz et al. [23] and Al-Saeed et al. [24] have found that the NWI is not a reliable indicator of these injuries on its own. A study by Uhorchak et al. showed an NWI of 0.18 [16], while another study by Domzalski et al. proposed a value of 0.24 [25]. In our study too, patients with ACL injury exhibited lower values of NWI with a mean value of 0.255 ± 0.025 as opposed to the control group with a slightly higher mean value at 0.267 ± 0.023. The application of NWI in avoiding ACL injuries was explained by Sourya et al. in their study where they proposed that young people with lower NWI and ACL tears were more likely to have injuries to their contralateral knee and should be advised of this danger at all times [21].

In our study, we were able to successfully establish a relation between narrow intercondylar NW and ACL tear. We also compared the NW between the two genders. Stijak et al., in their study, concluded that the NW in females was lower in comparison to males [15]. Similarly, in our study, lower values of NW were recorded in affected females than in the male case group. We also noted a mean NEW of 19.77 ± 2.79 in the control group as compared to a slightly higher value of 20.47 ± 3.06 in the group with ACL tear. A recent study by Fahim et al. noted a positive correlation of BW with ACL injuries; however in our study, both the groups were found to have almost similar measurements of BW [26].

With respect to the mean value of NWI, although our observations were similar to most studies, there may be discrepancies with certain studies owing to the fact that many previous studies used radiographs of the knee to measure morphometric parameters while we used MRI knee to assess the same. Some authors believe that the measurements from plain radiographs may not always be accurate as these measurements are subject to vary according to the variation in patient position, technique, magnification, and projection [27]. We recommend further studies on larger and differently cross-matched population groups to possibly assess baseline values for femoral NWI with the help of MRI scans.

However, this study is not without its limitations, and the fact that we did not ensure an approximately equal proportion of males and females may be our biggest limitation. Although we successfully matched the gender groups in terms of mean age and calculation method, an equal distribution of genders would have been ideal. The volume of the intercondylar notch is another important variable to consider, as some studies have reported that ACL-injured individuals have a smaller notch volume [28]. In our study, the bony morphology of the tibia was not considered, which is another highly recommended aspect for future studies.

## Conclusions

The study demonstrates notable distinctions in bony morphology between the groups with and without ACL injuries. We conclude that a narrow NW and NWI increase the risk of ACL injury in the tested population. Contact could occur between the medial side of the lateral femoral condyle and the ACL due to the narrowed space, potentially damaging the ligament. It is recommended that further research be conducted in order to accurately stratify patients and define a threshold value that will enable the measurement of the risk of an ACL injury in an MRI. Moreover, since bone morphology varies between populations, studies in the future should focus on developing indices that put into consideration a combination of various parameters rather than using absolute measures.
